# Clinical and economic impact of digital dashboards on hospital inpatient care: a systematic review

**DOI:** 10.1093/jamiaopen/ooaf078

**Published:** 2025-07-26

**Authors:** Enrico Coiera, Anastasia Chan, Kalissa Brooke-Cowden, Hania Rahimi-Ardabili, Nicole Halim, Catalin Tufanaru

**Affiliations:** Centre for Health Informatics, Australian Institute of Health Innovation, Macquarie University, Sydney, NSW 2109, Australia; Centre for Health Informatics, Australian Institute of Health Innovation, Macquarie University, Sydney, NSW 2109, Australia; Centre for Health Informatics, Australian Institute of Health Innovation, Macquarie University, Sydney, NSW 2109, Australia; Centre for Health Informatics, Australian Institute of Health Innovation, Macquarie University, Sydney, NSW 2109, Australia; Centre for Health Informatics, Australian Institute of Health Innovation, Macquarie University, Sydney, NSW 2109, Australia; Sydney Health Literacy Lab, University of Sydney, Sydney, NSW 2006, Australia; Centre for Health Informatics, Australian Institute of Health Innovation, Macquarie University, Sydney, NSW 2109, Australia

**Keywords:** hospitals, dashboard systems, database management systems, hospital stay, hospital costs

## Abstract

**Objective:**

Digital dashboards are used to monitor patients and improve inpatient outcomes in hospital settings. A systematic review assessed the impact of dashboards across five outcomes of hospital mortality, hospital length of stay (LOS), economic impacts, harms, and patient and carer satisfaction.

**Materials and Methods:**

Nine databases were searched from inception to May 2024. Studies were included if they reported primary quantitative research on dashboard interventions in hospital settings, were in English, and measured effectiveness for patients, caregivers, healthcare professionals or services. Data synthesis was performed via narrative review. Risk of bias was measured using Cochrane ROBINS-I and RoB 2.

**Results:**

We identified 5755 articles, and 70 met inclusion criteria. Of 20 findings reporting mortality (16 studies), five reported a decrease, whilst the majority (*n* = 15) found no significant change. LOS was reported across 43 findings (31 studies), with 28 reporting a reduction, an increase in five, and ten reporting no change. Of 21 findings (from 16 studies) reporting on harms, increases were observed in six, decreases in four, and no change in 11. Economic impacts were reported in 34 findings (31 studies), with the majority demonstrating reduced costs (*n* = 29), an increase in one, and no change in four. Eight findings (eight studies) reported on patient and carer satisfaction with care, with the majority (*n* = 6) demonstrating increased satisfaction, and two reporting no change.

**Discussion:**

Hospital dashboards do appear associated with either no change or a reduction in mortality, reduced costs, reduced LOS, and improved patient and caregiver satisfaction with care. Association with harms was equivocal.

**Conclusion:**

While there is evidence of potential benefits, actual impacts of hospital digital dashboard will likely be dependent on multiple local factors such as workflow integration.

## Background and significance

Dashboards are digital summaries that integrate critical clinical information for decision makers and often take advantage of data analytic methods.^1^ Digital dashboards can help monitor health service quality, safety, performance and support strategic management decisions.^2–5^ In hospitals, dashboards have been used to monitor and control clinical quality (e.g., mortality, surgical site infection rate), clinical efficiency (e.g., length of stay, wait time), patient safety (e.g., falls, medication incidents), patient reported measures and outcomes (e.g., inpatient satisfaction), organizational effectiveness (e.g., inpatient volume, emergency department volume), and staff performance (e.g., employee turnover, employee satisfaction).^6^

Whilst there is substantial variation in the design and use of dashboards, they may share the following characteristics:

- **Data**: A dashboard may integrate data from multiple sources (e.g., electronic patient records, pathology, pharmacy, imaging, or patient monitoring devices).
- **Analysis**: A dashboard may preprocess data (e.g., remove artefacts, smooth curves), or calculate higher order variables from raw data (e.g., aggregate metrics, risk scores, or performance indicators).
- **Visual presentation**: Dashboards may display selected subsets of data in a visual display designed to support decision-making, potentially in near real time, for example, using traffic light color schemes.
- **User interaction**: Dashboards may display alerts, prompts or reminders triggered by events in a data set to alert human users of potentially actionable events.

Claimed benefits for dashboards include the integration of large data sets into more easily interpretable visual formats for decision making, provision of notifications when measures deviate from predefined acceptable levels, prevention of adverse events, and improvement in the coordination of care.^7^ Dashboard use may be hindered by anxiety about performance surveillance, information overload, and a perceived lack of control of the performance measures monitored.^7^ Technical limitations that may hinder effective use include poor quality or nonstandardized data and ethical concerns related to the integration of multiple data sources.^7^

Despite the wide use of dashboards, to our knowledge, there has been no comprehensive systematic review of clinical and economic outcomes associated with dashboard use with hospital inpatients. One smaller systematic review of quality dashboards did identify a limited number of studies (*n* = 16) reporting clinical outcomes,^8^ with a focus on how such dashboards can improve nursing practice. Other systematic reviews have focused on process outcomes,^9–11^ or dashboard impact on quality and performance in specific clinical settings such as nursing,^7^^,^^8^^,^^12^ critical care,^13^^,^^14^ surgery,^15^ and patient safety.^16^ A challenge with all such studies is that local clinical characteristics may not be replicated in other settings, making it harder to directly compare the impact of digital health interventions like decision support systems or dashboards.^17^ Further, a dashboard will be just one element of a more complex socio-technical bundle of workflows and local practices.^18^

## Materials and methods

We conducted a systematic review of the literature to assess the impact of digital dashboards on patient, process, and financial outcomes in hospital settings. The protocol for this PRISMA^19^ compliant study is registered in PROSPERO (CRD42018106583).^20^

### Data sources and searches

We searched PubMed, CINAHL, MEDLINE and Embase via Ovid, Scopus, PsycINFO, COCHRANE Library, IEEEE Xplore, Web of Science (All Databases Search), and ACM Digital Library (Table S1). An independent librarian peer-reviewed the search strategy. The search was first conducted January 11, 2019-January 14, 2019, and updated on October 22, 2019, October 21, 2020, June 28, 2022, and February 13, 2023-February 14, 2023. A final search was conducted on May 15, 2024. Backward reference searching was conducted on all included studies.

### Study selection

We searched for primary quantitative research studies of digital dashboard interventions in hospitals (Table 1). In the initial two searches, two independent reviewers (CT and EC) screened titles and abstracts against inclusion criteria, and full text of included studies was retrieved. Reviewer differences were resolved through discussion. Titles, abstracts, and full text of studies retrieved in the 2020 search were screened by NH and EC in 2022, by KBC and EC in 2023, and in 2024 by KBC and AC.

**Table 1.** Study eligibility.

	Inclusion criteria
Population	Patient, healthcare staff, caregiver Any health condition
Intervention of interest	Electronic dashboard interventions for clinical, management or quality decisions in a hospital setting
Comparator	Any comparator or control
Outcome measure	Intervention effectiveness measured by: Reported benefits or harms for either patients (e.g., mortality, morbidity, quality of life, harms; timing and effect measures), caregivers (e.g., satisfaction with care), healthcare professionals (e.g., user satisfaction, usability), or healthcare services (e.g., process measures, healthcare output; timing and effect measures)
Study design	Primary quantitative research
Publication date	No limit
Language	English
	**Exclusion criteria**
Population	Not human
Intervention of interest	Not dashboard intervention
Study design	Not primary quantitative research
Language	Not English

### Data extraction and quality assessment

A standardized data extraction form^21^ was used focusing on mortality, patient and caregiver satisfaction with care, length of stay, costs, and harms. Four reviewers (CT, NH, KBC, and AC) independently extracted included study details for their search, with EC resolving conflicts.

Two reviewers evaluated risk of bias using ROBINS-I^22^ and RoB 2,^23^ with studies assigned a rating of “low,” “moderate,” “serious,” or “critical” for ROBINS-I and “low,” “some concerns,” and “high” for RoB 2.

### Data synthesis and analysis

Data synthesis used narrative methods as heterogeneity in intervention design, implementation, study design, and outcome measures precluded quantitative meta-analysis. Synthesis focused on intervention description, contextual factors (setting, study groups, data collection period), reported outcomes, and clinical domain.

## Results

We identified 5755 articles (Figure S1). After removing duplicates (*n* = 1334), 4421 articles were screened by title and abstract against inclusion and exclusion criteria. Of these, 559 full-text articles remained. After excluding records that could not be retrieved and further duplicates (*n* = 246), 313 articles were assessed for eligibility. In total, 70 articles were included in the review. Out of these studies, 50 quasi-experimental studies were assessed for bias with ROBINS-I, five randomized studies with RoB 2, and 15 studies were excluded from assessment as they were abstracts only (*n* = 14) or a PhD thesis (*n* = 1).

### Study characteristics

Of the 70 studies, 61 were quasi-experimental studies (predominantly before/after studies), six were randomized control trials, and three were observational studies, meaning there was some form of control for most studies. Of the 50 full-text studies assessed for risk of bias with ROBINS-I, one was “Critical” risk, 21 were “Serious,” and 28 were “Moderate” (Figures S3 and S4). Of the five studies assessed with RoB 2, three were rated “Some concerns” and two were rated “High” (Figures S5 and S6).

Interventions varied in design and implementation, but all aimed to monitor or improve patient outcomes through data visualization. They included dashboards to improve patient quality of care or safety (*n* = 21),^24–44^ organizational and staff performance dashboards (e.g., work efficiency) (*n* = 11),^45–55^ financial dashboards (e.g., cost-performance feedback) (*n* = 8),^56–63^ compliance dashboards (*n* = 7),^64–70^ clinical decision support system (CDSS) dashboards (*n* = 4),^71–74^ emergency department dashboards (*n* = 4),^75–78^ electronic health record (EHR)-based dashboards for monitoring or improving efficiency of Intensive Care Unit (ICU) data (*n* = 4),^79–82^ bedside computerized information systems (*n* = 3),^83–85^ dashboards for lab resource management (*n* = 3),^86–88^ antibiotic time-out dashboards (*n* = 2),^89^^,^^90^ bed management dashboards (*n* = 2),^91^^,^^92^ and a dashboard to increase patient participation (*n* = 1).^93^ Thirty-two of the dashboard interventions either leveraged data from the EHR or were embedded in an EHR system. Many dashboards provided real-time or near-time information and alerts, for example, using a traffic-light alert system.

The studies were undertaken in a variety of hospital settings: Inpatient (*n* = 16), ICU (*n* = 13), Surgery (*n* = 11), multiple settings (*n* = 10), Emergency Department (ED) (*n* = 4), Transfusion Services (*n* = 4), Hospital-wide (*n* = 3), Oncology (*n* = 2), Surgical ICU (*n* = 2), Pediatric ICU (*n* = 2), Infection Control/Prevention (*n* = 1), Cardiology (*n* = 1), and Acute care (*n* = 1).

The studies spanned publication years from 2003 to 2023 and were conducted in the United States (*n* = 50), Taiwan (*n* = 4), UK (*n* = 3), Australia (*n* = 2), Canada (*n* = 2), France (*n* = 1), Indonesia (*n* = 1), Iran (*n* = 1), Italy (*n* = 1), Korea (*n* = 1), Lebanon (*n* = 1), Netherlands (*n* = 1), Nigeria (*n* = 1), and Spain (*n* = 1).

Some studies reported multiple findings for one outcome due to differing clinical settings (e.g., ICU and hospital), subgroups (e.g., two treatments), and variations over time. As a result, the total number of findings for certain outcomes may exceed the number of studies reporting on that outcome.

### Dashboard impact on hospital mortality

Sixteen studies^24^^,^^25^^,^^28^^,^^29^^,^^35^^,^^38^^,^^55^^,^^68^^,^^70^^,^^73^^,^^77^^,^^81^^,^^83–85^^,^^90^ reported on patient mortality with 20 total findings (Tables S2 and S7). Of these, five findings reported a decrease in mortality (four results were statistically significant, one did not report statistical testing).^25^^,^^55^^,^^73^^,^^77^^,^^85^ No findings reported an increase in mortality. The majority of findings showed no statistically significant changes in mortality (*n* = 15),^24^^,^^25^^,^^28^^,^^29^^,^^35^^,^^38^^,^^68^^,^^70^^,^^81^^,^^83^^,^^84^^,^^90^ with three papers noting their study design was underpowered to detect changes.^24^^,^^29^^,^^84^ The findings reporting a decrease in mortality saw reductions varying from a minimum 19.4% reduction in risk-adjusted mortality^25^ to a 73% reduction for 30-day mortality rate.^55^

Several factors were identified as contributing to these reductions: improved data transparency enabling a surgerical department to better evaluate quality improvement efforts;^25^ the integration of an information technology bundle that allowed ICU specialists to provide 24/7 coverage using remote off-hours coverage;^85^ and facilitation of early clinical interventions through dashboard-usage which modified the progression of COVID-19-induced disease.^73^ In two studies (Birdas et al. and Staib et al.) the dashboard was implemented as one element of broader organizational change (implementing a new council of clinical operations and new chiefs of surgery) so outcomes could not be solely attributed to the dashboard.

### Dashboard impact on hospital length of stay

A total of 31 included studies reported on length of stay (LOS) with 43 total findings (Table S3).^24^^,^^25^^,^^27^^,^^28^^,^^30^^,^^34–36^^,^^39–42^^,^^44–46^^,^^48^^,^^50^^,^^54^^,^^61^^,^^66^^,^^68^^,^^70^^,^^77^^,^^78^^,^^81^^,^^83–85^^,^^87^^,^^90^^,^^93^ Nine of these studies did not conduct statistical analyses.^27^^,^^30^^,^^39^^,^^45^^,^^46^^,^^54^^,^^66^^,^^77^^,^^78^ A reduction in LOS was reported in 28 findings^24^^,^^25^^,^^27^^,^^28^^,^^30^^,^^34^^,^^39–41^^,^^45^^,^^46^^,^^48^^,^^50^^,^^54^^,^^66^^,^^68^^,^^70^^,^^77^^,^^78^^,^^81^^,^^83^^,^^84^^,^^90^ (16 findings were statistically significant and 11 did not report statistical significance). An increase in LOS was reported in five^28^^,^^35^^,^^46^^,^^61^^,^^68^ findings (with four statistically significant findings and one that did not report statistical significance). No significant change was reported in ten findings.^24^^,^^25^^,^^36^^,^^40^^,^^42^^,^^44^^,^^83^^,^^85^^,^^87^^,^^93^

Findings reporting a reduction in LOS ranged from a minimum of 0.28 days^34^ in average LOS to a maximum of 18.8 days.^84^ Reasons given for positive outcomes included dashboards allowing palliative care to be delivered sooner resulting in a shorter LOS,^28^ improved time to antibiotic administration for patients exhibiting signs of sepsis,^84^ and improved quality of care (including a reduction in medical errors and improved protocol compliance).^83^

The findings reporting an increase in LOS varied from a minimum of 0.1 days^61^ to a maximum of 8.5 days^28^ increase in median LOS. One study by Faber et al. reported negative outcomes due to an outlier patient with a 41-day LOS.^46^ In another study by Schnock et al. there was a correlation between high-dashboard-usage groups and longer LOS.^35^ However, the authors noted that patients with longer LOS had more opportunity to use the portal, hence these findings may not indicate a direct causal relationship between dashboard usage and longer LOS.

### Harms associated with dashboard use

A total of 16 included studies reported on harms with 21 total findings (Table S4).^25^^,^^26^^,^^29^^,^^32^^,^^33^^,^^44^^,^^48^^,^^58^^,^^59^^,^^63^^,^^64^^,^^67^^,^^71^^,^^74^^,^^77^^,^^89^ Statistical analysis was not performed or reported on for seven of these studies.^48^^,^^58^^,^^63^^,^^64^^,^^71^^,^^74^^,^^77^ An increase in harms was reported in six findings^33^^,^^58^^,^^63^^,^^67^^,^^74^^,^^89^ (three findings were statistically significant and three did not report statistical significance). A decrease was reported in four findings^25^^,^^33^^,^^44^^,^^67^^,^^71^ (three findings were statistically significant, and one did not report statistical significance). No change in harms was reported in 11 findings^26^^,^^29^^,^^32^^,^^48^^,^^59^^,^^64^^,^^77^^,^^89^ (three findings did not report statistical significance).

In the interventions with decreased harms, benefits included a 9.4% reduction in surgery complication rate,^25^ a 29% fall in adverse events,^44^ and a decrease in central line-associated bloodstream infection (CLABSI) rates from 2.6% to 0.7%.^33^ Harms reported with dashboards included an increase in inappropriate continuation of antibiotics (Vancomycin) from 0% to 5%,^89^ a decreased rate of hand hygiene adherence amongst physicians from 34.2% to 8.6%,^67^ and mild hypoglycaemia in 20 patients (11.2% of patients).^74^

### Economic impacts of dashboard use

A total of 31 included studies reported on costs with 34 total findings (Table S5).^25^^,^^27^^,^^37^^,^^40^^,^^41^^,^^43^^,^^47–49^^,^^52^^,^^53^^,^^55–63^^,^^65^^,^^72^^,^^74^^,^^75^^,^^79–82^^,^^86^^,^^88^^,^^92^ No statistical analysis was performed or reported for 16 of these studies.^27^^,^^41^^,^^47^^,^^49^^,^^52^^,^^56^^,^^58^^,^^60^^,^^62^^,^^65^^,^^72^^,^^75^^,^^80^^,^^86^^,^^88^^,^^92^ A reduction of costs was reported in the majority of findings (*n* = 29)^25^^,^^27^^,^^37^^,^^40^^,^^41^^,^^43^^,^^47–49^^,^^52^^,^^55^^,^^56^^,^^58–63^^,^^65^^,^^72^^,^^75^^,^^79–82^^,^^86^^,^^88^^,^^92^ (13 findings were statistically significant, 16 did not report statistical significance). A statistically significant increase in costs was reported in one finding,^40^ and no significant change in three findings.^53^^,^^57^^,^^74^

Studies reporting cost reductions showed annual hospital savings ranging from a minimum of US$160 000 to a maximum of US$10.7 million.^62^ In the latter study, use of the dashboard workflow allowed a perioperative leadership team, surgeons, and supply chain personnel to standardize procedures and eliminate unnecessary items adding to costs, and reduced wastage in surgical or medical supplies such as red blood cells. The study by Niday et al. conducted at Johnson City Medical Centre used a financial dashboard as part of a multifaceted strategy to eliminate high-cost contract nursing, allowing the department to save $7 million annually.^58^ Studies with positive outcomes also reported savings in staff time, ranging from 132-198 hours of work saved per year^80^ to 11,200 hours saved per year.^41^

### Dashboard impact on patient and carer satisfaction

A total of eight included studies reported on patient and caregiver satisfaction with care, with eight total findings (Table S6).^31^^,^^44^^,^^51^^,^^53^^,^^58^^,^^69^^,^^76^^,^^91^ No statistical analysis was performed for five of these studies.^31^^,^^51^^,^^58^^,^^76^^,^^91^ The majority of findings (*n* = 6) reported an increase in parent and carer satisfaction after dashboard implementation (three findings were statistically significant results, and three did not report statistical significance).^44^^,^^51^^,^^53^^,^^69^^,^^76^^,^^91^ No findings reported a decrease in satisfaction, and two findings reported no change (both did not test or report statistical significance).^31^^,^^58^

Reasons given for positive outcomes included clinicians being able to see information such as patient wait times more easily^76^ and web-based checklists allowing patient preferences and concerns to be more systematically addressed.^44^ The study by Hartzler et al. reported no change in satisfaction and the authors speculated that a lack of improvement in patient satisfaction may have resulted from a “ceiling effect,” as scores were near maximum both before and after the dashboard intervention.^31^

## Discussion

A review of the 70 included studies indicated that digital dashboards are widely used across diverse hospital settings with the intention of improving the process of care delivery and patient outcomes. This review demonstrates clear potential for beneficial impacts of dashboards across process and patient outcomes:

- Of the 20 total findings (in 16 studies) reporting on mortality, five reported a decrease in mortality, and the remaining 15 findings found no significant change. Observed mortality reductions could be substantial. In a surgical setting, separate studies identified a 19.4% reduction in risk-adjusted mortality^25^ and a 73% reduction for 30-day mortality rate.^55^
- Patient length of stay in hospital, a proxy for service quality and effectiveness, was reduced in the majority of findings reporting this outcome (*n* = 28), with ten reporting no change, and an increase in five. LOS reductions from a minimum of 0.28 days^34^ in average LOS for a maternity dashboard, up to a maximum of 18.8 days for a bedside sepsis intervention.^84^
- A similar number of findings reported an increase in patient harms (*n* = 6) and a decrease (*n* = 4), with no change in 11 findings.
- A reduction in costs was reported in 29 of the studies undertaking an economic analysis, with an increase in costs one study, and no change in four others. Cost reductions ranged from US$160 000 (FY2014) in annual hospital operating room expenditure for gastric bypass surgeries^47^ to US$10.7 million (FY2014) in the cost of total knee arthroplasty in another hospital using a peri-operative dashboard.^62^
- Eight findings reported on patient and carer satisfaction with hospital care, with the majority (*n* = 6) demonstrating increased satisfaction, and two studies reported no change.

### Strengths and weaknesses of the study

This review focuses on core clinical process and economic outcomes of digital dashboards across a wide variety of settings and tasks. The volume of literature reviewed is substantial and prior reviews^7–16^ have been smaller in size and have not examined this broad set of important outcomes.

Although we identified and analysed 70 studies and the majority had some form of control (including “before/after” studies), they were of variable quality, and some were at high risk of bias, for example because they were single arm. Many were conducted as quality improvement studies, where local impact is assessed and generalizability to other settings is less clear. Further, quality improvement studies might indicate that hospitals have more mature quality improvement processes and resources compared to sites which do not undertake such activities. There is a risk of publication bias in the results presented, where negative outcomes are either not reported or are harder to publish, although we do identify reports of harm in 16 of the included studies. Consequently, whilst we can report clear overall patterns of impact, generalizing these patterns to new hospital settings will need to account for local factors such as dashboard use, case mix, workflows, technology environments, and study designs.

### Implications

This review provides good evidence that significant and positive outcome improvements are achievable in principle with digital dashboards in hospitals. It cannot however provide clear evidence for what might be achieved when a digital dashboard is implemented in a new setting. Further, only six of the studies were randomized control trials, and the bulk of the remainder were before/after studies. While this allows us to have confidence in the associational effects reported, it would require more experimental bench tests under controlled conditions, along with real world RCTs, to be undertaken before we unpack the causal chain between dashboard use and dashboard impact.

As is the case with all digital interventions in clinical settings, without capturing and assessing these local confounding factors, we cannot provide a full explanation for any outcome achieved. For example, poor workflow integration may lead to a digital dashboard being inconvenient to use, resulting in low uptake by clinicians. Furthermore, a study that focuses only on downstream outcomes of dashboard use might detect no endpoint changes (e.g., mortality rates) but this does not provide evidence for lack of dashboard effectiveness in general and may only reflect the local workflow.

This study does help tease apart which clinical hospital departmental settings have demonstrated improvements and which have not. Unpacking these differences further will be an important focus for future research. New tools such as the IMPISCO framework provide a mechanism for comparing studies to assist in assessing how replicable results are from one study to another.^94^ Discovering and comparing variations in impact of similar interventions in different settings should also be instructive in terms of dashboard design and implementation, by identifying those contextual factors most associated with improved outcomes.

Conceiving of outcome measures for digital interventions along a value chain starting with user interactions with the technology and ending with clinical outcomes can allow a richer interpretation of cause and effect (Figure S2).^94^ Future studies should, therefore, conduct more ecosystemic analyses, by reporting on key process measures that reflect the intervention in use, alongside final outcomes like mortality, cost-effectiveness, or length of stay.

## Conclusions

Digital dashboards are widely used across a variety of hospitals settings and tasks, and this comprehensive systematic review provides evidence that, when an intervention is implemented well, significant improvements in critical clinical, process, and financial outcomes are possible. The existence of multiple confounding factors related to a specific hospital service and the fit between dashboard design and implementation, and clinical work, mean that local outcome improvements are likely to always be contingent on these local circumstances.

## Supplementary Material

ooaf078_Supplementary_Data

## Data Availability

Template data collection forms, data extracted from included studies, and data used for all analyses are available upon request from the authors.
