# Supplementary material for: Clinical and economic impact of digital dashboards on hospital inpatient care: a systematic review
Source: JAMIA Open. 2025 Jul 26;8(4):ooaf078. doi: 10.1093/jamiaopen/ooaf078 (PMC12296400; doi:10.1093/jamiaopen/ooaf078)
Supplement: ooaf078_Supplementary_Data [file ooaf078_supplementary_data.zip › JAMIAO Supplementary File.docx]

**Supplementary Online Content**

**eTable 1.** Search strategy for Medline via Ovid (1946 to October 4, 2024)

**eFigure 1.** PRISMA Flow Diagram

**eFigure 2.** Examples of Evaluation Metrics at Different Stages of the Information Value Chain

**eTable 2.** Mortality Changes Associated with Digital Dashboard Use in 16 Studies

**eTable 3.** Length of Stay (LOS) Changes Associated with Digital Dashboard Use in 30 Studies

**eTable 4.** Changes in Harms Associated with Digital Dashboard Use in 16 Studies

**eTable 5.** Changes in Costs Associated with Digital Dashboard Use in 31 Studies

**eTable 6.** Changes in Patient and Carer Satisfaction Associated with Digital Dashboard Use in 8 Studies

**eTable 7.** Acronyms for Quantitative Results Tables

**eFigure 3.** Summary Plot of Risk-of-Bias Domains as per ROBINS-I Tool

**eFigure 4.** Traffic Light Plot of Risk-of-Bias Domains as per ROBINS-I Tool

**eFigure 5.** Traffic Light Plot of Risk-of-Bias Domains as per Cochrane RoB 2 Tool (Individual RCT)

**eFigure 6.** Traffic Light Plot of Risk-of-Bias Domains as per Cochrane RoB 2 Tool (Cluster RCT)

**eTable 1.** **Search strategy for Medline via Ovid (1946 to October 4, 2024)**

| **#** | **Query** |
| --- | --- |
| 1 | hospitals/ or hospitals, general/ or hospitals, high-volume/ or hospitals, low-volume/ or hospitals, private/ or hospitals, public/ or hospitals, rural/ or hospitals, satellite/ or hospitals, teaching/ or hospitals, university/ or hospitals, urban/ or tertiary care centers/ |
| 2 | dashboard.mp. [mp=title, book title, abstract, original title, name of substance word, subject heading word, floating sub-heading word, keyword heading word, organism supplementary concept word, protocol supplementary concept word, rare disease supplementary concept word, unique identifier, synonyms, population supplementary concept word, anatomy supplementary concept word] |
| 3 | hospital*.mp. [mp=title, book title, abstract, original title, name of substance word, subject heading word, floating sub-heading word, keyword heading word, organism supplementary concept word, protocol supplementary concept word, rare disease supplementary concept word, unique identifier, synonyms, population supplementary concept word, anatomy supplementary concept word] |
| 4 | 1 or 3 |
| 5 | 2 and 4 |
| 6 | (hospital* and dashboard*).tw. |
| 7 | 5 and 6 |
| 8 | limit 7 to yr="2019 - 2023" |
| 9 | limit 8 to yr=2023-current |

**eFigure 1. PRISMA Flow Diagram**

**PRISMA Flow Chart – Effectiveness of Hospital Dashboard Interventions**

Reports not retrieved:

Other (Duplicates): (n = 5)

Misc (multiple categories): (n = 241)

**(n = 246)**

Records excluded:

**(n = 3862)**

Records screened (by title and abstract):

**(n = 4421)**

Reports sought for retrieval

**(n = 559)**

**Identification**

Records identified from database searches

Databases: (n = 3338)^a^, (n = 404)^b^, (n = 689)^c^, (n = 480)^d^, (n = 528)^e^, (n = 266)^f^

**(n = 5705)**

Additional records identified through other sources: **(n = 50)**

Records removed *before screening*:

Duplicate records removed:

**(n = 1334)**

**Screening**

Reports excluded:

Not the Study design of interest: (n = 14)

Not the Setting of interest: (n = 5)

Not the Intervention of interest: (n = 77)

Not the Outcomes of interest: (n = 5)

**(n = 101)**

Reports assessed for eligibility

**(n = 313)**

Total studies included in systematic review

**(n = 212)**

Quantitative studies included in narrative synthesis

**(n = 70)**

**Included**

**Figure 1. Search Strategy for papers**

a – Initial search conducted Jan 2019

b – Search conducted Oct 2019

c – Search conducted Oct 2020

d – Search conducted June 2022

e – Search conducted February 2023

f – Search conducted May 2024

**eFigure 2. Examples of Evaluation Metrics at Different Stages of the Information Value Chain.** Focusing entirely on outcomes without assessing up-stream variables that may shape outcome limits generalisability of outcome evidence.

**eTable 2. Mortality Changes Associated with Digital Dashboard Use in 16 Studies**

| **Authors, Year, Country** | **Setting** | **Intervention Date and Number of Participants** | **Intervention Description** | **Representative Reported Outcomes and Representative Results** |
| --- | --- | --- | --- | --- |
| Anderson BJ, et al. 2019; USA | ICU | Pre-intervention, 1 May ‒ 25 Sep 2016 (n=221)  Post-intervention, 26 Sep 2016 ‒ Feb 2017 (n=236) | Implementation of the Awakening and Breathing Coordination (ABC) Application – an EHR-based dashboard and text alert intervention which aimed to promote patient sedation minimisation and ventilator liberation. | NONE: No statistically significant difference in ICU mortality [n, %] was found between patients admitted before [75, 31.8%] and after [78, 35.3%) the intervention was implemented (p=0.70).  NONE: No statistically significant difference in hospital mortality [n, %] was found between patients admitted before [93, 42.1%] and after [94, 39.8%) the intervention was implemented (p=0.48). |
| Birdas TJ, et al. 2019; USA | Surgery | Intervention period: Jan 2017 – June 2018  Before: (2016, n=39,576),  After (2017-18, n=45,004) | Structural reorganisation initiative which included the implementation of a dashboard to improve quality of care and surgical outcomes. | DECREASE: Risk-adjusted mortality showed a statistically significant reduction in mortality index by 19.4% [pre-intervention: 1.17; post-intervention: 0.94] (**p=0.01**) in Group 1 (Surgery).  NONE: No statistically significant differences in mortality index were found in Group 2 (Other Surgery).  NONE: No statistically significant differences in mortality index were found in Group 3 (Non-Surgery). |
| Cox CE, et al. 2018; USA | ICU | Intervention period: 6 Jun –  30 Sep 2016  Intervention (n=14)  Control A (n=25)  Control B (n=39) | Implementation of an EMR-integrated web app platform, Palliative Care Planner (PCplanner), which aimed to improve the delivery of ICU-based palliative care by identifying unmet patient family needs. | NONE: A lower hospital mortality [n, %] was observed in the intervention group [n=4, 29%] compared to both control groups [Control A: n=14, 56%; Control B: n=18, 46%] but this was not statistically significant. |
| Fletcher GS, et al. 2018; USA | Inpatient | Intervention period: 9 Feb – 29 Jun 2009  Intervention (n=4914)  Control (n=4851) | Inclusion of a novel user dashboard within an existing EMR system aiming to trigger rapid response action by providing real-time feedback on acute patient symptoms. | NONE: No statistically significant difference was found in the incidence rate ratio [IRR] of deaths occurring on general medical-surgical wards between periods when the dashboard was on (intervention group) compared to when the dashboard was off (control group) [0.96] (p=0.89). |
| Jung AD, et al. 2018; USA | Surgical ICU | Before: (2016 – 2017, n=23)  After: (2017, n=7) | The display of sepsis screen scores (SSS) on automated bedside clinical surveillance visualisation systems aimed to improve patient monitoring and clinical outcomes. | NONE: No statistically significant difference in hospital mortality was found between pre- and post-intervention groups (no details provided). |
| Levesque E, et al. 2015; France | ICU | Before: (2004 – 2005, n=662)  After: (2005 – 2006, n=735) | Implementation of a bedside intensive care information system (ICIS) which aimed to document patient-monitoring data and reduce the risk of human error. | NONE: No statistically significant difference in mortality rate [n, %] was found between patients admitted before [n=74, 11.2%] and after [n=71, 9.6%] the intervention was implemented (p=0.35). |
| McCambridgeM, et al. 2010; USA | ICU | Intervention period: 1 Jan – 30 Sep 2004    Intervention (n=959)  Control (n=954) | Implementation of an EMR system aiming to increase off-site intensivist coverage to 24h per day. | DECREASE: Both hospital and ICU mortality rates (%) were significantly lower in the intervention group (14.7% and 11.5%, respectively) compared to the control group (21.4% and 15.8%, respectively) (**p<0.001** and **p=0.006**, respectively). |
| Miller RS, et al. 2010; USA | ICU | Intervention period: 1 Jan 2006 – 30 Apr 2008  Pre-intervention (n=895)  Post-intervention 2007 (n=827)  Post-intervention 2008 (n=231) | Implementation of several staff and clinical reforms, including a ventilator-associated pneumonia (VAP) dashboard, which aimed to monitor and increase compliance with infection control best practice. | NONE: No statistically significant difference in hospital mortality [n, %] was found between patients admitted pre- [n=130, 16%] and post-intervention [2007: n=94, 14%; 2008: n=21, 13%] (p=0.54). |
| Olchanski N, et al. 2017; USA | ICU | Intervention period: 2011 –  2013  Pre-intervention, 2010 (n=983)  Post-intervention, 2014 (n=856) | Implementation of an EMR intervention (ProCCESs AWARE) which aimed to monitor ICU patient data such as clinical, task-specific, and administrative information. | NONE: No statistically significant difference was found in hospital mortality rate [pre-intervention: 7.2%; post-intervention: 6.1% (p=0.20)]  NONE: No statistically significant difference was found in ICU mortality rate [pre-intervention: 4.6%; post-intervention: 3.4% (p=0.33)]. |
| Pettit NN, et al. 2019; USA [Conference Poster Abstract only] | Inpatient | Pre-intervention, 1 Apr – 31 Jul 2017 (n=94)  Post-intervention, Oct –Dec 2018 (n=521) | Implementation of an automated antibiotic time out (ATO) dashboard alerting clinicians to patients who have received at least 48 hours of antibiotic therapy, aiming to improve efficiency of post-treatment care. | NONE: No statistically significant difference was found in inpatient mortality rate [n, %] between patients admitted before [n=7, 7.4%] and after [n=64, 12.3%] the intervention (p=0.16). |
| Schnock K, et al. 2022, USA | Inpatient (Oncology and neurology) | Intervention period: Mar – May 2018  High usage group (n=27)  Moderate usage group (n=92)  Low-usage group (n=69) | Implementation of a patient safety dashboard (Safety Advisor) which aimed to provide real-time patient safety information and tailored educational content, and to assess impact on health outcomes. | NONE: A decrease in hospital mortality rate was observed in the high-portal-usage group (high-usage group [n = 27; 0.5%], moderate-usage group [n = 92; 2.1%], and low-usage group [n = 69; 1.1%]) but this was not statistically significant. |
| Shaw SJ, et al. 2015; USA | ICU - Paediatric | Intervention start date: Jul 2013  Group 1, Apr – May 2013 (n=150)  Group 2, Aug 2013 (n=150)  Group 3, Nov 2013 (n=150) | Implementation of an online, integrated platform monitoring patient compliance with the PICU Safety Bundle, which aimed to identify six common low-priority concerns for patient safety. | NONE: Across all three groups there were no statistically significant differences found in neither PICU mortality rate [n, %]- [Group 1: n=6, 4%; Group 2: n=3, 2%; Group 3: n=2, 1% (p=0.41)], nor Paediatric Index of Mortality (PIM2) predicted mortality [% (IQR)]- [Group 1: 0.72 (0.16-1.00); Group 2: 0.71 (0.15-0.92); Group 3: 0.75 (0.19-1.10) (p=0.15)]. |
| Staib A, et al. 2017; Australia | ED | Intervention start date: early 2014 | Development and implementation of an ED inpatient interface (EDii) dashboard which aimed to monitor efficiency and quality of care of ED inpatient processes. | DECREASE: From pre- to post-intervention emergency admission mortality rate decreased from 2.3% to 1.0%, respectively. [Paper did not report any detailed quantitative results or statistical analyses to support this finding]. |
| Verma A, et al. 2023; USA [Conference Abstract only] | Cardiology | Intervention period:  Sep 2021 – Dec 2021  Intervention group (n=150)  Control group (n=150) | Implementation of a Heart Failure (HF) dashboard to monitor and improve outpatient HF management for veterans. | NONE: A dashboard-based clinical intervention did not improve the optimization potential score (OPS) or secondary outcomes of hospitalization and all-cause mortality for veteran patients with heart failure. There was a no significant difference between the intervention group and the control group (optimization potential score (OPS), 2.9; SD=2.1 versus OPS, 2.6, SD=2.1); adjusted mean difference 0.3 (95% CI, -0.1 to 0.7). |
| Vizcaychipi MP, et al. 2020; UK | ICU; General wards | Intervention period: 12 Apr – 21 May 2020  Pre-intervention (n=535)  Bridge-intervention (n=204)  Post-intervention (n=200) | Implementation of a novel decision tool on patient EMRs aiming to detect Thromboembolic and Cytokine storm risk using a near real-time Traffic-light system (TraCe-Tic). | DECREASE: Age- and sex-adjusted probability of death [OR (95% CI)] was significantly lower in those to post-intervention [0.22 (0.17-0.27)] compared to pre-intervention [0.33 (0.30-0.37)] (**p<0.001**). |
| Zygourakis CC, et al. 2017; USA | Surgery | Before (2014, n=186),  After (2015, n=63) | Multi-departmental hospital-based study involving the electronic delivery of monthly surgeon scorecards to decrease surgical supply and procedural costs. | DECREASE: A statistically significant improvement in 30-day post-discharge mortality [OR (95% CI)] from pre- to post-intervention was observed in the intervention group compared to the control group [0.27 (0.11-0.67)] (**p=0.005**). |

**eTable 3. Length of Stay (LOS) Changes Associated with Digital Dashboard Use in 31 Studies**

| **Authors, Year, Country** | **Setting** | **Intervention Date and Number of Participants** | **Intervention Description** | **Representative Reported Outcomes and Representative Results** |
| --- | --- | --- | --- | --- |
| Anderson BJ, et al. 2019; USA | ICU | Pre-intervention: 1 May ‒ 25 Sep 2016 (n=221)  Post-intervention: 26 Sep 2016 ‒ Feb 2017 (n=236) | Implementation of the Awakening and Breathing Coordination (ABC) application – an EHR-based dashboard and text alert intervention which aimed to promote patient sedation minimisation and ventilator liberation. | DECREASE: A statistically significant reduction in ICU LOS [adjusted HR; 95% CI] post-intervention [1.31; 1.03–1.67] (**p = 0.027**).  NONE: No statistically significant difference was found in hospital LOS [adjusted HR; 95% CI] post-intervention [1.15; 0.90–1.47] (p = 0.26). |
| Becker B, et al. 2021; USA | Inpatient (Hospital Medicine) | Pre-intervention: Jul 2017 to Jun 2018  Post-intervention: Jul 2018 to Jun 2019 | Implementation of a monthly feedback report comprising an individualised dashboard with peer comparison report (rank order list) which aimed to improve quality patient outcomes. | DECREASE: LOS index was stated to have improved, however, provider-level LOS data was not available owing to changes in the data extraction and reporting within the healthcare system. [Paper did not report any detailed quantitative results or statistical analyses to support this finding]. |
| Birdas TJ, et al. 2019; USA | Surgery | Intervention period: Jan 2017 – Jun 2018  Before (2016, n=39,576),  After (2017-18, n=45,004) | Structural reorganisation and establishment of dashboard featuring quality elements of patient care. | DECREASE: Statistically significant improvements in risk-adjusted LOS index were found in Group 1 [relative reduction by 6.6% from pre-(1.11) to post-(1.04) intervention, **p=0.002**].  DECREASE: : Statistically significant improvements in risk-adjusted LOS index were found in Group 2 [relative reduction by 5.3% from pre-(1.06) to post-(1.00) intervention, **p=0.01**].  NONE: No statistically significant difference was found in risk-adjusted LOS index for Group 3. |
| Clark KW, et al. 2014; Australia | Inpatient (Medical, Surgical and Maternity Wards) | Intervention start date: Feb –  Nov 2011 | Implementation of ward-specific electronic patient journey boards (EPJBs) which aimed to enhance patient safety and continuity of care through improvements in quality of patient flow. | DECREASE: Following the intervention, reductions in average LOS for inpatients were experienced for multiple diagnostic related groups (DRG). In the 14 medical ward pilot sites, between 57% and 92% of medical wards recorded a reduction in average LOS. [Paper did not report any detailed quantitative results or statistical analyses to support this finding].  DECREASE: In the seven surgical ward pilot sites, between 57% and 100% of surgical wards recorded a reduction in average LOS. [Paper did not report any detailed quantitative results or statistical analyses to support this finding].  DECREASE: In the four maternity ward pilot sites, between 50% and 100% of maternity wards recorded a reduction in average LOS. [Paper did not report any detailed quantitative results or statistical analyses to support this finding]. |
| Cox CR, et al. 2018; USA | ICU | Intervention period: 6 Jun –  30 Sept 2016  Intervention: (n=14)  Control A: (n=25)  Control B: (n=39) | Implementation of an EMR-integrated web app platform, Palliative Care Planner (PCplanner), to improve the delivery of ICU-based palliative care by proactively identifying unmet patient family needs. | DECREASE: A statistically significant difference in LOS was shown through a lower [mean (SD); median (IQR)] number of hospital LOS days in the intervention group [20.5 (9.1); 17.5 (14.8-26.7) days] compared to control group B [29.7 (16.1); 29.0 (17.0-36.0) days] (**p<0.05**).  INCREASE: A statistically significant difference was found in total ICU LOS [mean (SD); median (IQR)] number of days between the intervention group [16.1 (8.1); 15.5 (9.5-25.3) days] and control group A [11.5 (12.9); 7.0 (4.3-13.0) days] (**p<0.05** for rank-sum test). |
| Dykes PC, et al.; 2017; USA | ICU | Pre-intervention:1 Jul 2013 –  8 Jun 2014 (n=881)  Post-intervention: 1 Jul 2014 –29 May 2015 (n=904) | Implementation of a web-based Patient Engagement Communication and Technology (PROSPECT) program with patient portal which aimed to improve patient safety and outcomes. | NONE: No statistically significant difference was found in medical ICU LOS [mean days], between pre-intervention [4.9 days] and post-intervention [5.0 days] (p=0.61). |
| Faber B, et al.; 2012; USA | Inpatient | Intervention period: 1 Nov 2010 – 30 Nov 2011 | Design and implementation of novel visual communication metrics included as part of a hospital-wide initiative, which aimed to increase proactivity of hospital operational processes. | DECREASE: Initial 4 months showed a decrease in number of patients with a LOS greater than 6 days. [Paper did not report any detailed quantitative results or statistical analyses to support this finding].  INCREASE: Total number of patients with a LOS over 6 days increased between April and June. [Paper did not report any detailed quantitative results or statistical analyses to support this finding]. |
| Gnanasekaran G, et al. 2017; USA [Conference Abstract only] | Inpatient | [Intervention period and study group(s) not reported]  (n=45) | Implementation of an electronic dashboard intervention (PRIDE), which aimed to improve patient quality of care and safety of elderly patients at risk of delirium and hospital readmissions. | DECREASE: A reduction in LOS was found between pre- (7.7 days) and post- (5.8 days) intervention. [Paper did not report any detailed quantitative results or statistical analyses to support this finding]. |
| Hansen TL, et al. 2023; Canada  [Conference abstract only] | Inpatient | Data collection periods: Unknown | Implementation of a Stroke Dashboard to visually display real-time data on multi-site stroke units. | DECREASE: After the Dashboard was implemented, there was an 8% increase in admission to stroke units, a 1.9d reduction in length of stay, 10% increase in Alpha-FIM completion within 3d and reduced wait times for tests. [Paper did not report any detailed quantitative results or statistical analyses to support this finding]. |
| Hechenbleikner EM, et al. 2022; USA | Surgery (Metabolic and bariatric) | Pre-intervention: May 2019 –  Apr 2020 (n=193)  Post-intervention: May 2020-Jul 2021 (n=278) | Implementation of a real-time data support dashboard (Tableau), checklist, and an updated Enhanced recovery protocol (ERP bundle) aimed to assess compliance and evaluate 30-day patient outcomes. | DECREASE: A reduction in LOS was found between pre- (2.0 days) and post- (1.7 days) intervention. [Paper did not report any detailed quantitative results or statistical analyses to support this finding]. |
| Hester G, et al. 2019; USA | ED; Inpatient | Intervention period: Oct 2015- Apr 2018  Patients discharged home from ED [baseline n=2,035; intervention n=3,965]  Inpatients [baseline n=843; intervention n=1,485] | Implementation of a visual analytics dashboard aiming to improve quality and clinical process outcomes in patients with bronchiolitis. | DECREASE: In the cohort of patients discharged home from ED, a statistically significant reduction in ED LOS [mean (95% CI) hours] was found between baseline [2.9 (2.7-3.1) hours] and intervention [2.6 (2.5-2.6) hours] (**p<0.001**).  NONE: In the cohort of inpatients, no statistically significant difference in inpatient LOS [mean (95% CI) days] was found between baseline [2.3 (2.2-2.4) days] and intervention [2.6 (2.4-2.8) days] (p=0.07). |
| Javier-DesLoges J, et al. 2020; USA [Conference Abstract only] | Surgery | Pre-intervention: 2016 –2017  Post-intervention: 2018 –2019  Total n= 4,798 | Implementation of a peri-operative dashboard aiming to improve surgery outcomes through blinded individual and group performance surgeon scorecards. | DECREASE: A statistically significant reduction in mean LOS (mean ± SD days) was found from pre- (4.75 ± 3.82 days) to post- (4.21 ± 2.48 days) intervention (**p<0.01**). |
| Jung AD, et al. 2018; USA | Surgery | Intervention start date: Jul 2017  Pre-intervention: Jul 2016 –  June 2017 (n=23)  Post-intervention: Jul 2017 – Sep 2017 (n=7) | The display of sepsis screen scores (SSS) on automated bedside clinical surveillance visualisation systems aimed to improve patient monitoring and clinical outcomes. | DECREASE: A statistically significant difference in LOS was shown by a reduction in average numbers [mean ± SE] of ICU LOS days, from pre- [19.1 ± 3.3 days] to post- [7.6 ± 2.5 days] intervention (**p<0.01**).  DECREASE: A statistically significant difference in hospital LOS days [mean ± SE] from pre- [29.6 ± 4.3 days] to post- [10.8 ± 3.1 days] intervention (**p<0.01**). |
| Kai-Hsuan Y, et al. 2020; Taiwan | ICU | Intervention period: Jul 2016 –  Jul 2017  Intervention: (n=1,101)  Control: (n=1,147) | Implementation of an automated technology electronic dashboard-ICU (TED-ICU) system aiming to provide real-time feedback and improve ICU patient quality of care. | DECREASE: A statistically significant difference was found in adjusted ICU LOS [mean number of days (95% CI)] of [-1.78 (-2.42 to -1.15)], resulting in a lower number of ICU LOS days [mean (SD)] in the intervention group [5.53 (4.99) days] compared to the control group [7.26 (9.57) days] (**p<0.001**). |
| Leikin S, Mohamed A, et al. 2019; Country Not specified [Conference Abstract only] | ICU | Pre-intervention: Nov 2017 –  Jan 2018 (n=172)  Post-intervention: Nov 2018 –  Jan 2019 (n=126) | Implementation of a novel integrated electronic health record tool aiming to monitor and improve patient mobility goals. | NONE: No statistically significant difference was found in hospital LOS [mean number of days] between pre- [35.26 days] and post- intervention [26.84 days] (p=0.17). |
| Levesque E, et al. 2015; France | ICU (Liver ICU) | Intervention start date: Apr 2005  Pre-intervention: Jan 2004 –  Mar 2005 (n=662)  Post-intervention: Jun 2005 – Aug 2006 (n=735) | Implementation of a bedside intensive care information system (ICIS), aiming to electronically document patient-monitoring data and reduce the risk of human error. | DECREASE: A statistically significant decrease was found in ICU LOS [mean ± SD days] between pre-(8.5 ± 15.2 days) and post-(6.8 ± 12.9 days) intervention (**p=0.048**).  NONE: No statistically significant difference was found in hospital LOS [mean ± SD days] between pre-(27.7 ± 34.6 days) and post-(28.6 ± 33.3 days) intervention (p=0.79). |
| McCambridge M, et al. 2010; USA | ICU | Intervention period: 1 Jan – 30 Sep 2004  Intervention group: (n=959)  Control group: (n=954) | Implementation of a health information technology bundle and remote intensivist coverage (HITB-RIC) intervention - an EMR system aiming to increase off-site intensivist coverage to 24h per day. | NONE: No statistically significant difference was found in either the mean number of ICU LOS days [pre-intervention: 4.1 days; post-intervention: 3.8 days (p=0.88)] or hospital LOS days [pre-intervention: 9.2 days; post-intervention: 9.2 days (p=0.83)]. |
| Miller RS, et al. 2010; USA | ICU (Trauma ICU) | Intervention period: 1 Jan 2006 – 30 April 2008  Study groups:  2006 (n=895)  2007 (n=827)  2008 (n=231) | Implementation of several staff and clinical reforms, including a ventilator-associated pneumonia (VAP) dashboard, which aimed to monitor and increase compliance with infection control best practice. | INCREASE: A statistically significant increase was found in hospital LOS [median (IQR) days] from 2006 [7 (3-13) days] to 2007 [10 (5-17) days], and to 2008 [9 (5-15) days] **(p<0.001).**  DECREASE: A statistically significant difference in ICU LOS [median (IQR) days] was found from 2006 [3 (1-8 days)] to 2007 [4 (2-8) days] and to 2008 [3 (2-7) days] (**p=0.01**). |
| Meidani Z, et al. 2021: Iran | Inpatient (Neurology) | Pre-intervention: 15 Mar ‒ 15 Jun 2019 (n=3038)  Post-intervention: 16 Jun ‒ 15 Sep 2019 (n=1840) | Implementation of an automated audit and feedback dashboard system which aimed to reduce laboratory test ordering by neurology residents by providing individualised and peer-comparison data. | NONE: No statistically significant difference was found in hospital LOS [mean±SD] between pre- [6.66±5.31] and post-intervention [6.46±5.81] (p=0.467). |
| Olchanski N, et al. 2017; USA | ICU | Intervention start date: Jul 2012  Pre-intervention: 2010 (n=983)  Post-intervention: 2014 (n=856) | Implementation of Patient Centred Cloud-based Electronic System: Ambient Warning and Response Evaluation (ProCCESs AWARE) to monitor ICU patient data. | DECREASE: A statistically significant reduction in LOS [mean ± SD] was shown through a decrease in ICU LOS [pre-intervention: 4.1±4.9 days; post-intervention: 2.5 ± 3.2 days (**p<0.0001**)].  DECREASE: A statistically significant reduction was also found in hospital LOS [pre-intervention: 12.1±14.6 days; post-intervention: 8.2 ± 9.1 days (**p<0.0001**)]. |
| Patel H, et al. 2017; USA | Inpatient | Pre-intervention: Jul 2012 – Jun 2013 (n=6,572)  Intervention: Jun 2013 – Jun 2014 (n=6,179)  Post-intervention: Jul 2014 – Jun 2015 [n not provided] | Implementation of a quality improvement initiative, including a web-based dashboard, which aimed to increase the rate of early discharge of inpatients. | DECREASE: Across all three study periods, statistically significant differences in LOS were shown through a reduction in the annual average LOS (number of midnights patient spends during admission) [baseline: 5.88 intervention: 5.85; post-intervention: 5.60 (**p<0.05**)], and reduction in LOS index (risk-adjusted observed-to-expected LOS) [baseline: 1.16; intervention: 1.18; post-intervention: 1.10 (**p<0.05**)]. |
| Pettit NN, et al. 2019; USA [Conference Poster Abstract only] | Inpatient | Pre-intervention: 1 Apr – 31 Jul 2017 (n=94)  Post-intervention: 1 Oct – 31 Dec 2018 (n=521) | Implementation of an automated antibiotic time out (ATO) dashboard to alert clinicians to patients who have received at least 48 hours of antibiotic therapy, aiming to improve efficiency of post-treatment care. | DECREASE: A statistically significant reduction in LOS [median number of days] was found from pre-(18 days) to post-(15 days) intervention (**p=0.01**). |
| Robinson JR, et al. 2018; USA | Surgery (OR) | Pre-intervention: 1 Jan - 30 Jun 2016 (n=110)  Post-intervention: 1 Oct 2016 – 31 Mar 2017 (n=106) | Implementation of an automated Tableau dashboard which monitored real-time OR supply cost data and evaluated clinical and cost outcomes. | INCREASE: A statistically significant difference in LOS was shown through an increase in time from admission order to discharge order [median (IQR) days] from pre-intervention [1.1 (0.8-1.5) days] to post-intervention [1.2 (0.9-1.6) days] (**p=0.023**). |
| Schnock K, et al. 2022; USA | Inpatient (Oncology and Neurology) | Intervention period: Mar – May 2018  High-usage group: (n=27)  Moderate-usage group: (n=92)  Low-usage group: (n=69) | Implementation of a patient safety dashboard (Safety Advisor) which aimed to provide real-time patient safety information and educational content, and to assess impact on health outcomes. | INCREASE: A statistically significant difference in LOS [mean (SD), days] was observed between high-portal-usage group [8.3 (12.0)], moderate-portal usage group [6.8 (10.0)] and low-portal usage group [7.1 (7.3)] (**p=0.01**). |
| Schnock KO, et al. 2019; USA | Inpatient (Acute care) | Intervention period: Dec 2016 – May 2018  Intervention: (n=1,619)  Control: (n=1,219) | Implementation of a web-based bedside patient portal displaying educational content, which aimed to increase overall patient activation. | NONE: No statistically significant difference was found in LOS [mean (SD) time units unspecified] between the intervention group [8.85 (8.99)] and control group [8.61 (8.44)] (p=0.63). |
| Shaw SJ, et al. 2015; USA | ICU (Paediatric) | Intervention start date: Jul 2013  Group 1: Apr – May 2013 (n=150)  Group 2: Aug 2013 (n=150)  Group 3: Nov 2013 (n=150) | Implementation of an online, integrated platform monitoring patient compliance with the PICU Safety Bundle, which aimed to identify six common low-priority concerns for patient safety. | DECREASE: A statistically significant reduction in PICU LOS [median (IQR) days] was found in Group 2 [1 (1-3) days] compared to both Group 1 [2 (1-5) days] and Group 3 [2 (1-5) days] (**p=0.005**). |
| Soh JY, et al. 2019; Republic of Korea | Surgery | Intervention period: Apr – May 2018  Intervention (n=22)  Control (n=22) | Implementation of a mobile app-based dashboard intervention (Go-breath) which aimed to improve patients’ post-operative use of incentive spirometers (IS). | NONE: No statistically significance difference found in LOS [mean (SD) time units (resumed days)] between the intervention [10.8 (1.6) days] and control [10.4 (1.0) days] groups (p=0.57). |
| Spektor M, et al. 2008; USA [Conference Abstract only] | ED | Intervention start date: mid-Apr 2007  Pre-intervention: Jan – Apr 2007  Post-intervention: May – Jul 2007 | Implementation of an automated electronic dashboard which aimed to monitor patient time intervals in the ED. | DECREASE: Statistically significant reductions in ED LOS [median average time (units not stated)]] were found in Group 1 [pre-intervention: 13:00; post- intervention: 10:12 (**p<0.05**)] and Group 2 [pre-intervention: 3:40; post-intervention: 3:24 (**p<0.05**)]. |
| Staib A, et al. 2017; Australia | ED | Intervention start date: early 2014 | Development and implementation of an ED inpatient interface (EDii) dashboard aiming to monitor efficiency and quality of care of ED inpatient processes. | DECREASE: From pre- to post-intervention ED LOS decreased from 7.2 to 3.8 h, respectively. [Paper did not report any detailed quantitative results or statistical analyses to support this finding]. |
| Stone-Griffith S, et al.; 2012; USA | ED | Intervention start date: 2006  Data collection periods:  2007 | Development and implementation of an ED Dashboard and Reporting Application aiming to improve quality of care and workflow systems. | DECREASE: From 2007 to 2010, there was a reduction in LOS [median mins] by 10.5%, from 200 to 179 mins, respectively. [Paper did not report any detailed quantitative results or statistical analyses to support this finding]. |
| Weiner J, et al. 2015; USA | ED; Hospital-wide | Pre-intervention: Jan 2012  Post-intervention: Jan 2014 | Development and implementation of visual web-based dashboards (iDashboard) providing real-time feedback on operational and staff performance metrics. | DECREASE: Following the intervention, reductions in average monthly LOS [mins] were found in both Acute Track patients [pre-intervention: 343.4 mins; post-intervention: 289.0 mins] and Lean Track patients [pre-intervention: 167.4 mins; post-intervention: 108.8 mins]. [Paper did not report any detailed quantitative results or statistical analyses to support this finding]. |

**eTable 4. Changes in Harms Associated with Digital Dashboard Use in 16 Studies**

| **Authors, Year, Country** | **Setting** | **Intervention Date and Number of Participants** | **Intervention Description** | **Representative Reported Outcomes and Representative Results** |
| --- | --- | --- | --- | --- |
| Ayad M, et al. 2017; USA [Conference Abstract only] | ICU | Intervention period: Jul 2014 – Feb 2016  Pre-intervention: (n=4050)  Post-intervention: (n=15,533) | Implementation of a data visualisation dashboard in high-risk ICUs to identify heparin dosing delays and monitor dose administration discrepancies. | NONE: Whilst the rate of Heparin Induced Thrombocytopenia [(HIT) per 1000 hospital days] increased from pre- [1.23] to post-intervention [1.29], authors conclude that the HIT rate remained unchanged (presumably non-significant). [Paper did not report any detailed quantitative results or statistical analyses to support this finding]. |
| Birdas TJ, et al. 2019; USA | Surgery | Intervention period: Jan 2017 – June 2018  Before: (2016, n=39,576)  After: (2017-18, n=45,004) | Structural reorganisation initiative which included the implementation of a dashboard to improve quality of care and surgical outcomes. | DECREASE: A statistically significant reduction in Complication rate (%) was observed in Group 1 (-9.4%) [pre-intervention to 7.9 post-intervention: 7.1] (**p=0.02**). Complications included postoperative and medical complications.  NONE: No statistically significant reduction in Complication rate (%) was observed in Group 2 (+19.4%) [pre-intervention to 5.3 post-intervention: 6.4] (p=0.12).  NONE: No statistically significant reduction in Complication rate (%) was observed in Group 3 (+6.5%) [pre-intervention to 3.5 post-intervention: 3.8] (p=0.2). |
| Boord JB, et al. 2007; USA | Surgical intensive care unit (SICU) | Pre-intervention: 3 Nov – 23 Nov 2004  Post-intervention: 7 Dec 2004 – 24 Jan 2005 | Implementation of an integrated glycaemia control protocol within the care provider order entry (CPOE) system. | NONE: No difference was detected in the incidence of severe hypoglycaemia (≤ 40 mg/dl) [%], from pre-intervention [0. 2%] to post-intervention [0.2%]. |
| Dykes PC, et al.; 2017; USA | ICU | Pre-intervention:1 Jul 2013 –  8 Jun 2014 (n=881)  Post-intervention: 1 Jul 2014 – 29 May 2015 (n=904) | Implementation of a web-based Patient Engagement Communication and Technology (PROSPECT) program with patient portal which aimed to improve patient safety and outcomes. | NONE: No statistically significant difference was found in harms, or medical ICU LOS [mean days], between pre-intervention [4.9 days] and post-intervention [5.0 days] (p=0.61). |
| Fletcher GS, et al. 2018; USA | Inpatient Acute Care Wards | Intervention period: Feb – Jun 2009  Intervention: (n=4914)  Control: (n=4851) | Inclusion of a novel user dashboard within an existing EMR system aiming to trigger rapid response action by providing real-time feedback on acute patient symptoms. | NONE: No statistically significant difference was found in harms, or the number of cardiopulmonary arrests, between periods when the dashboard was on (n=11) and when the dashboard was off (n=7) (p=0.43). |
| Graber CJ, et al.; 2015; USA | ICU | Data Collection Period:  Pre-intervention: 16 Apr – 18 Oct 2012  Post-intervention: 19 Apr – 18 Oct 2013 | Implementation of an antimicrobial time-out program (for vancomycin and piperacillin-tazobactam) with an antimicrobial dashboard, designed to reduce inappropriate antimicrobial therapy | INCREASE: Harm, measured as the inappropriate continuation of antibiotics [n, % guideline discordant continuations], was significantly increased after course treatment using Vancomycin [pre-intervention: 0, 0%, post-intervention: 7, 5%] (**p=0.002**).  NONE: There was no statistically significant difference after course treatment using Piperacillin-tazobactam [pre-intervention: 2, 2%, post-intervention: 9, 9%] (p=0.06). |
| Javier-DesLoges J, et al. 2020; USA [Conference Abstract only] | Surgery | Pre-intervention: 2016 –2017  Post-intervention: 2018 –2019  Total n= 4,798 | Implementation of a peri-operative dashboard aiming to improve surgery outcomes through blinded individual and group performance surgeon scorecards. | NONE: Following implementation of the intervention, there was no difference in post-operative adverse events. [Paper did not report any detailed quantitative results or statistical analyses to support this finding]. |
| Lenglet A, et al. 2019; Nigeria | Inpatient/Hospital-wide | Intervention period: 23 Apr – 25 May 2018  Pre-intervention: (n = 686)  Post-intervention: (n = 673) | Implementation of a multimodal strategy including an interactive dashboard as a quality improvement project to improve hand hygiene adherence in health care workers. | INCREASE: There was an increase in harm, or decreased rate of hand hygiene adherence [%] among physicians at one hospital from pre-intervention [34.2%] to post-intervention [8.6%] (**p<0.001**).  DECREASE: However, there was a decrease in harm, or increased rate of hand hygiene adherence [%] among nurses from pre-intervention [11.5%] to post-intervention [61.4%] (**p<0.001**). |
| Mccoy AB, 2010; USA [PhD Thesis] | Inpatient/ICU | Pilot Study: Feb – May 2010  Intervention: (n=200)  Control: (n=196) | Implementation of a web-based surveillance tool (dashboard) designed to assist clinical pharmacists in monitoring patients with acute kidney injury (AKI) with the intention of reducing ADEs and potential CDS failures. | NONE: Following the intervention, there were no significant improvements in the occurrence, preventability, and severity of pADEs and ADEs. A total of 44 (22.45%) control and 45 (22.61%) intervention cases experienced a pADE (RR =1.01; ([Reviewer Note: we presumed RR is Relative Risk]), and 59 (4.53%) control and 63 (4.52%) intervention medication orders had an associated pADE (RR=1.00). A total of 32 (16.33%) control and 24 (12.00%) intervention cases experienced an actual ADE (RR=0.74), and 36 (2.76%) control and 30 (2.15%) intervention medication orders had an associated actual ADE (RR=0.78). |
| Niday P, et al. 2012; USA | Hospital-wide | Pre-intervention: 2008  Post-intervention: 2009 | Implementation of a multifaceted intervention, including a financial dashboard, to improve nurse scheduling in acute care settings and reduce nurse staffing expenditure. | INCREASE: Although considered to promote cost-efficiency in hospital operations, the dashboard intervention eliminated the use of contract nursing staff and could be perceived as producing harm to contract nurses as a result of subsequent job losses. [Paper did not report any detailed quantitative results or statistical analyses to support this finding]. |
| Offodile AC, et al. 2020; USA | Surgery | Intervention period: 1 Jan – 31 Dec 2018  Total n=2853 | Implementation of a custom dashboard (Know Your Costs) as a cost feedback tool to reduce intraoperative supply costs (ISC). | NONE: After implementation of the intervention, no statistically significant difference was detected in incidence of postoperative complications [n, %] between 2016 [n=711, 5.1%], 2017 [n=1023, 5.1%] and 2018 [n=1119, 5.3%]. |
| Pageler NM, et al. 2014; USA | ICU (Paediatric) | Intervention period: 1 May – 31 Aug 2011 | Implementation of an EMR checklist attached to a patient safety and quality dashboard to improve compliance with catheter-care bundles and reduce the rate of central line-associated bloodstream infections (CLABSI). | DECREASE: There was a decrease in compliance [%] with best practice (insertion) bundle element, from pre-intervention [67%] to post-intervention [62%] (**p=0.001**).  INCREASE: There was increased compliance with daily documentation of line necessity from 30% to 73% (**p=0.001**), increased compliance with dressing changes from 87% to 90% (**p=0.003**), increased compliance with cap changes from 87% to 93% (**p=0.001**), and increased compliance with port needle changes from 69% to 95% (**p=0.001**). |
| Simpao AF, et al. 2015; USA | Hospital-wide | Intervention period: Jan 2011 – Jan 2014 | Implementation of a CDSS-based dashboard as a quality improvement initiative aiming to reduce the volume of clinically irrelevant alerts and decrease alert fatigue in pharmacists. | DECREASE: Following implementation of the interventions, no serious safety events (SSE) were reported. The hospital medication SSE rate per 10,000 adjusted patient days decreased from baseline to post-intervention [0.18 – 0.08]. [Paper did not report any detailed quantitative results or statistical analyses to support this finding]. |
| Smith GA et al. 2015; UK | Hospital transfusion services | Intervention start date: 1 Dec 2012  Pre-intervention: Dec 2011 – July 2012  Post-intervention: Dec 2012 – July 2013 | Multifaceted intervention involving analysis of RBC wastage via dashboard, which was also used to compare wastage before and after wastage reduction interventions. | INCREASE: An increase in staff (Biomedical Scientists) time [per unit issued] was found from pre- to post-intervention [3.4 to 3.8 per unit], assessed as equivalent to the cost of 18units/pa (at a cost conversion of £130/RBC unit) totalling £2,340/pa. [Paper did not report any detailed quantitative results or statistical analyses to support this finding]. |
| Staib A, et al. 2017; Australia | ED | Intervention start date: early 2014 | Development and implementation of an ED inpatient interface (EDii) dashboard which aimed to monitor efficiency and quality of care of ED inpatient processes. | NONE: Following implementation of the intervention, no harms, or adverse safety signals [number of Rapid Response Team activations within the first 24h of ED admission] were detected. [Paper did not report any detailed quantitative results or statistical analyses to support this finding]. |
| Vogelzang M, et al. 2005; Netherlands | ICU (Surgical) | Intervention period: 1 Jan – 1 May 2005  Pre-intervention: Nov – Dec 2004 (6 weeks) (n=unspecified)  Post-intervention: 1 Jan – 1 May 2005 (n=179) | Implementation of Glucose Regulation for Intensive care Patients (GRIP) – a CDSS designed to assist with glucose control by intensive insulin therapy, and provide recommendations for insulin delivery and timing of blood glucose testing. | INCREASE: Harm, measured as mild hypoglycaemia (<3.5 mM), occurred in 11.2% of all 179 patients. [Paper did not report any detailed quantitative results or statistical analyses to support this finding]. |

**eTable 5. Changes in Costs Associated with Digital Dashboard Use in 31 Studies**

| **Authors, Year, Country** | **Setting** | **Intervention Dates and Number of Participants** | **Intervention Description** | **Representative Reported Outcomes and Representative Results** |
| --- | --- | --- | --- | --- |
| Batley NJ, et al. 2011; Lebanon | ED | Voluntary anonymous Staff Questionnaire administered Feb – Mar 2009 | Implementation of an EMR-based ED Dashboard designed to track patients and improve other ED processes. | DECREASE: Following intervention, there was a potential reduction in costs, or 3-person-weeks of labour saved, per year. Additionally, an estimated 900h of time potentially saved in laboratory ordering by the ED, per year. [Paper did not report any detailed quantitative results or statistical analyses to support this finding]. |
| Birdas TJ, et al. 2019; USA | Surgery | Intervention period: Jan 2017 –  Jun 2018  Before (2016, n=39,576),  After (2017-18, n=45,004) | Structural reorganisation initiative which included the development and implementation of a dashboard aiming to improve quality of care and surgical outcomes. | DECREASE: A statistically significant reduction in risk-adjusted direct cost index, was observed in Group 1 (11.8%) from pre- (0.99) to post-intervention (0.87) (**p=0.0001**), or an estimated increase in savings of $2,300 per surgical admission.  DECREASE: A statistically significant reduction in risk-adjusted direct cost index (-4.8%) observed in Group 3 from pre-intervention (0.96) to post-intervention (0.92) (**p=0.008**).  NONE: No statistically significant difference in risk-adjusted direct cost index was observed for Group 2, with an increase of 0.1% from pre- (0.95) to post-intervention (0.96) (p=0.95). |
| Carmona-Cejudo JM, et al. 2012; Spain | Hospital Laboratory | Intervention start date: 2008 | Implementation of a web-based laboratory information management system (DB4US) designed to optimise the use of lab resources | DECREASE: At 6 months post-intervention, a reduction in costs was demonstrated through a 78% reduction in the usage of a specific laboratory reagent. [Paper did not report any detailed quantitative results or statistical analyses to support this finding]. |
| Clark KW, et al. 2014; Australia | Inpatient (Medical, Surgical and Maternity Wards) | Intervention start date: Feb –  Nov 2011 | Implementation of ward-specific electronic patient journey boards (EPJBs) which aimed to enhance patient safety and continuity of care through improvements in quality of patient flow. | DECREASE: Following intervention, there was a reduction in cost, or increased savings of staff time ranging from 20 mins to 2.5 hr per staff member per shift. [Paper did not report any detailed quantitative results or statistical analyses to support this finding]. |
| Ducatman AM, et al. 2017; USA | Inpatient and outpatient; Emergency Medicine; Urgent Care | Pre-intervention: Jul 2011 – Sep 2012  Intervention: Sep 2012 – Dec 2013  Post-intervention: Jan 2014 – Mar 2015 | Implementation of a quality improvement intervention with dashboard feedback to reduce low-value laboratory tests for diagnosis of Myocardial Infarction (MI) and improve guideline compliance. | DECREASE: Following intervention, annual laboratory cost savings at an academic hospital was over $635,000 USD. [Paper did not report any detailed quantitative results or statistical analyses to support this finding]. |
| Frosini F, et al. 2016; Italy | Surgery; OR | Intervention period: 2012-2013  Post-intervention: Jan 2012 – Dec 2012 (n=1054 surgeries)  Post-intervention: Jan 2013 – Dec 2013 (n=1065 surgeries) | Development of a web-based real-time operational dashboard for monitoring the efficiency of OT usage and safety according to key performance indicators (KPIs). | DECREASE: Following intervention, reduction in costs were demonstrated through increases in average annual savings of time (29.52 mins per surgery) and annual monetary saving for 2013 ($343 362,60). An increase in OT usage reported as 5% (over the 2-year study period). [Paper did not report any detailed quantitative results or statistical analyses to support this finding]. |
| Gunaratne K, et al. 2016; Canada | Surgery | Pre-intervention: Jan – May 2014 (n=114)  Post-intervention: Sep – Dec 2014 (n=88) and Jan – Apr 2015 (n=107) | Implementation of an email accessible customised Surgeon Cost Report Card aiming to provide cost-performance feedback to increase awareness of costs. | DECREASE: Progressive reductions in surgical costs [mean (SD) per surgery,] were observed from pre-intervention [$3038 ($305)] to Time period 1 post-intervention [$2859 ($391); -5.9%], and at Time period 2 post-intervention [$2827 ($402); -6.9%]. Potential annual savings for (n=293) procedures at [$2492 ($410)] equate to (USD)$160,000. [Paper did not report any detailed quantitative results or statistical analyses to support this finding]. |
| Hester G, et al. 2019; USA | ED; Inpatient | Intervention 1 Oct 2015 – 30 Apr 2018  Patients discharged home from ED (baseline n=2,035; intervention n=3,965)  Inpatients (baseline n=843; intervention n=1,485) | Implementation of a visual analytics dashboard aiming to improve quality and clinical process outcomes in patients with bronchiolitis. | DECREASE: A statistically significant reduction in ED charges [mean ratio] was observed from baseline [1.0] to time of implementation [0.86] **(p<0.001).**  INCREASE: A statistically significant increase in inpatient charges [mean ratio] was observed from baseline [1.0] to time of implementation [1.14] (**p=0.01**). |
| Javier-DesLoges J, et al. 2020; USA [Conference Abstract only] | Surgery (Urology) | Pre-intervention: 2016-2017  Post-intervention: 2018-2019  Total n= 4,798 (4-year period) | Implementation of a peri-operative dashboard aiming to improve surgery outcomes through blinded individual and group performance surgeon scorecards. | DECREASE: There was a statistically significant reduction in total cost of hospital stay [mean ± SD] from baseline [$7,147 ± 67.21] to post-intervention [$6,278 ± 5.76] (**p<0.01**). |
| Kai-Hsuan Y, Kao et al. 2020; Taiwan | ICU | Intervention period: Jul 2016 – Jul 2017  Intervention: (n=1,101)  Control: (n=1,147) | Implementation of an automated dashboard (TED-ICU) system aiming to provide real-time feedback and improve ICU patient quality of care. | DECREASE: Following the intervention, there was a reduction in cost, or increased time savings of ICU healthcare staff work time [1121 hours saved/month], and physician and nurses’ care handover time [11,200 hours saved/year]. Following the intervention, a cost saving of US$155,425 due to reduction in LOS was realised within 6 months (total saving of $277,425 - $122,000 for implementation costs). [Paper did not report any detailed quantitative results or statistical analyses to support this finding]. |
| Kingeter A, et al. 2018; Country Not specified [Conference Abstract only] | ICU | Data Collection Periods: Alternating 12-week periods of dashboard access with 14-week and 12-week Control periods (no access) | Implementation of an interactive Tableau dashboard designed to provide cost transparency to ICU providers for patients under their care | NONE: No statistically significant difference was found in costs [median overall charges per patient per day] between the Cost-Transparency periods [$9,825] compared to the control periods [$10,018] (p=0.21). |
| Lai CH, et al. 2022; Taiwan | ICU (Surgical) | Intervention period: 26 Apr – 18 Jul 2021  Intervention: (n=78 MDRs)  Control: (n=91 MDRs) | Implementation of EMR-based i-Dashboard, an information management platform designed to improve efficiency of data gathering for multi-disciplinary rounds (MDRs) and facilitate information exchange during MDRs. | DECREASE: A statistically significant reduction in cost, or staff time [median (IQR)] spent gathering pre-round data per patient, was demonstrated from pre-intervention [10.4 (9.1-11.8)] and post-intervention [4.6 (3.5-5.8)] minutes (**p<0.001**), demonstrating a reduction of 5.8 (95% CI 5.2-6.4) minutes. |
| Lo YS, et al. 2014; Taiwan | Infection control unit | Intervention period: 1 Nov 2010 – 22 April 2011  Study groups:  eCBSIS: (n=35)  iHAUTISIS: (n=69) | Implementation of an EMR-based integrated hospital-associated urinary tract infection surveillance information system (iHAUTISIS) dashboard, designed to improve work efficiency for infection control professionals. Comparison of intervention with existing electronic culture-based surveillance information system (eCBSIS). | DECREASE: There was a reduction in hospital staff time cost [marginal mean seconds saved per patient, SD] by 73.2 s from when eCBSIS was implemented [187.54 ± 84.87 s] to when iHAUTISIS was implemented [114.26 ± 57.43 s]. [Paper did not report any detailed quantitative results or statistical analyses to support this finding]. |
| Mayfield J, et al. 2013; USA [Conference Abstract only] | Infection control unit; ICU; Oncology | Intervention period: Not stated | Implementation of CA-BSI and VAP dashboards designed to streamline ICU and Oncology Infection Prevention data | DECREASE: Following intervention, there was a reduction in cost, or increased savings of total hospital staff time [cumulated hours per month] spent on packaging patient data [total of 60-90 hours] across 11 patient care areas [11-16.5 hours saved per month per care area]. [Paper did not report any detailed quantitative results or statistical analyses to support this finding]. |
| Niday P, et al. 2012; USA | Hospital-wide | Pre-intervention: 2008  Post-intervention: 2009 | Implementation of a multifaceted intervention, including a financial dashboard, to improve nurse scheduling in acute care settings and reduce nurse staffing expenditure. | DECREASE: Following the intervention, reductions in nursing staff costs resulted in increased annual financial savings of > $7.2 million. [Paper did not report any detailed quantitative results or statistical analyses to support this finding]. |
| Olchanski N, et al. 2017; USA | ICU | Intervention start date: Jul 2012  Pre-intervention: 2010 (n=983)  Post-intervention: 2014 (n=856) | Implementation of Patient Centred Cloud-based Electronic System: Ambient Warning and Response Evaluation (ProCCESs AWARE) - an EMR intervention to monitor ICU patient data. | DECREASE: There was a statistically significant reduction in total mean charges [USD] for hospital stay per hospitalisation by 30% [-$43,745] from pre-intervention [$149,593] to post-intervention [$103,383] (**p<0.0001**). |
| Offodile AC, et al. 2020; USA | Surgery | Intervention period: 1 Jan – 31 Dec 2018  Total n=2853 | Implementation of a custom dashboard (Know Your Costs) as a cost feedback tool to reduce intraoperative supply costs (ISC). | DECREASE: There was an unadjusted reduction in mean surgical costs of selected procedures from baseline ($2,681.46) to post-intervention ($1,909,53), and a statistically significant reduction of 20% in overall surgical costs equating to hospital savings of $883,942 across the study period (**p<0.0001**). |
| Pickering BW, et al. 2015; USA | ICU | Data Collection Period: 26 Mar – 3 Jun 2012 | Implementation of an EMR-based dashboard (AWARE) designed to support bedside clinical information management in the ICU and improve efficiency of data management. | DECREASE: A statistically significant reduction in staff time [mean (95% CI)] spent gathering pre-round data per patient, was demonstrated from pre-intervention [12.0 (10.0 to 15.0) mins] to post-intervention [9.0 (7.3 to 11.0) mins] corresponding to 0.74-fold change in time spent (**p=0.03**). |
| Pizzini GA, et al. 2019; USA | Anaesthesia department; Pharmacy | Intervention start date: July 2016  Pre-intervention: Jul 2015 – Jun 2016  Post-intervention: Jul – Nov 2016 | Implementation of intervention comprised of a change management process and anaesthesia dashboard designed to track drug usage patterns and decrease unnecessary pharmaceutical costs. | DECREASE: Following intervention, there was a reduction in mean pharmaceutical costs of ephedrine and epinephrine by 67% from pre-intervention ($38,000 per month for ephedrine alone) to post-intervention (<$8,000 for both agents). [Paper did not report any detailed quantitative results or statistical analyses to support this finding]. |
| Robinson JR, et al. 2018; USA | Paediatric Surgery; OR | Pre-intervention: 1 Jan – 30 Jun 2016 (n=110)  Post-intervention: 1 Oct 2016 – 31 Mar 2017 (n=106) | Implementation of an automated Tableau dashboard which monitoring OR supply cost data. | DECREASE: There was a statistically significant reduction in OR costs [median (IQR)] by 56% from pre-intervention [$884 ($705-$1025)] to post-intervention [$388 ($182-$776)] (**p<0.001**). |
| Rocchio BJ; 2016; USA | Surgery | Pre-intervention: FY2013  Post-intervention: FY 2014-15 | Implementation of a perioperative clinical dashboard designed to provide a holistic view of surgical procedures in real-time and reduce OR supply costs. | DECREASE: Following intervention, reduction in costs were observed through increases in total cost savings of >$1.2 million for total knee arthroplasty procedures in FY 2014, and >$10.7 million for perioperative services in FY 2015. [Paper did not report any detailed quantitative results or statistical analyses to support this finding]. |
| Rosow E, et al. 2003; USA | ED; OR; Inpatient | Intervention start date: Apr 2001 | Implementation of a real-time Bed Management Dashboard (BMD) to streamline and optimise the processes of patient admission, transfer, and discharge. | DECREASE: Following the intervention, there was a $200,000 reduction in annual hospital expenses by $200,000. [Paper did not report any detailed quantitative results or statistical analyses to support this finding]. |
| Sexton P, et al. 2022; USA | PACU (Post-anaesthesia Care Unit) | Pre-intervention: 2019  Post-intervention: Jan 2020 – May 2021 | Implementation of an EMR-based nursing patient dashboard with verbal report, aiming to provide an efficient and evidence-based hand-off communication process to reduce PACU and OR boarder rates. | DECREASE: Following the intervention, though there was no statistically significant difference in average PACU boarder rates, an observed reduction of 34 min, representing a monthly saving of approximately $15,755 USD (at $171 per 15-minute-stay). |
| Smith GA et al. 2015; UK | Hospital transfusion services | Intervention start date: 1 Dec 2012  Pre-intervention: Dec 2011 – July 2012  Post-intervention: Dec 2012 – July 2013 | Multifaceted intervention involving analysis of RBC wastage via dashboard, which was also used to compare wastage before and after wastage reduction interventions. | DECREASE: A statistically significant reduction in cost or red blood cell (RBC) wastage [%] was found for adult RBC (excluding group AB and B positive) from pre- to post-intervention [5.28% to 2.54%] (**p<0.0001**), which is a reduction of approximately 52%. A total saving of 115 units/pa equates to financial savings of £14,950 (at a cost conversion of £130/RBC unit). |
| Stadler JG, et al. 2016; USA | Inpatient | Data Collection Periods:  Year 1  Years 2 & 3 (projected) | Implementation of EHR-based visualisation dashboards designed to improve sepsis outcomes and prevent 30-day re-admissions. | DECREASE: Following the intervention, there was a reduction in cost, or increase in total time savings in Year 1 of 289 hours of staff working hours saved. A statistically significant reduction in time spent per client project was found with automation vs manual analysis of sepsis in Year 1, with a reduction of [t(73) = 4.97] (**p<0.001**).  No statistically significant difference in time savings was found with automation vs manual analysis of 30-day readmissions in Year 1, however, projected time savings in Years 2 and 3 when compared with manual analysis of 37 hrs and 100 hrs was found to be a reduction [t(17) = 2.16] (**p<0.05**). |
| Staples S, et al. 2020; UK | Hospital transfusion services | Intervention start dates:  2014 (education and feedback intervention)  2017 (hospital-wide dashboard intervention + emails) | Implementation of CDSS for blood ordering dashboard, in addition to quarterly summary emails, aimed to reduce inappropriate transfusion orders and blood product costs. | DECREASE: There was a 26% reduction in costs of total blood products found from pre-intervention ($5,123,875) to post-intervention ($3,783,414). [Paper did not report any detailed quantitative results or statistical analyses to support this finding]. |
| Varghese S, et al. 2020; USA  [Conference Abstract only] | Oncology | Intervention period: not specified | Implementation of an EMR-based dashboard to facilitate the Claim Request process for specialty medications requiring Prior Authorisation. | DECREASE: Savings measured as money refunded to patients. Up to 16% of patients had money refunded as a result of timely CR [Claim Request] submission. [Paper did not report any detailed quantitative results or statistical analyses to support this finding]. |
| Vogelzang M, et al. 2005; Netherlands{Vogelzang, 2005 #45} | ICU (Surgical) | Intervention period: 1 Jan – 1 May 2005  Pre-intervention: Nov – Dec 2004 (6 weeks) (n=unspecified)  Post-intervention: Jan – May 2005 (n=179) | Implementation of Glucose Regulation for Intensive care Patients (GRIP) – a CDSS with user interface designed to assist with glucose control by intensive insulin therapy. | NONE: No statistically significant difference in cost, or staff time [median (IQR)] spent on glucose control per shift, was found between pre-intervention [10 (6-12) mins] and post-intervention [10 (10-15) mins] (p=0.13). |
| Wang JC, et al. 2019; Taiwan  [Conference Abstract only] | Inpatient | Intervention Period: Jan 2017 – Dec 2018  Pre-intervention: (n=10)  Post-intervention: (n=10) | Implementation of an App linked to a web-based dashboard to facilitate communication between patients and ERAS staff members and improve efficiency and efficacy of care. | NONE: No statistically significant difference was found in cost or time savings [mean staff working hours] from pre-intervention [9.2±1.3 hours] to post-intervention [8.2±1.1 hours] (p=0.66). |
| Woo JS, et al. 2019; USA | Hospital transfusion services | Intervention period: not specified | Implementation of a real-time web-based dashboard designed to improve the workflow of blood product inventory management and monitoring of blood product containers (coolers). | DECREASE: Post-implementation, an estimated total of 256 minutes of staff time was saved per day. Using a rate of $55/hr (the wage for a blood bank technician) an estimated saving of $235/day or $7040/month, was reported. [Paper did not report any detailed quantitative results or statistical analyses to support this finding]. |
| Zygourakis et al. 2017; USA | Surgery | Intervention period: 1 Jan – 31 Dec 2015  Intervention: (n=63)  Control: (n=186) | The OR Surgical Cost Reduction (OR SCORE) project was a multi-departmental hospital-based study involving the electronic delivery of monthly surgeon scorecards which aimed to decrease surgical supply and procedural costs. | DECREASE: A statistically significant reduction in costs, or increase in annual surgical cost savings [mean (95% CI)], was detected in the intervention group [9.95% (3.55% to 15.93%)] over the one-year period (p=0.003). The intervention group demonstrated a lower rate of spending [mean (95% CI)] on surgical supplies [−$71 (−$195 to $54) per case] compared to the control group [$178 ($126 to $231) per case] over the one-year period. This resulted in an annual cost-savings total of $836,147 in the intervention group compared to an increase of $3,073, 647 in annual costs for the control group. |

**eTable 6. Changes in Patient and Carer Satisfaction Associated with Digital Dashboard Use in 8 Studies**

| **Authors, Year, Country** | **Setting** | **Intervention Date and Number of Participants** | **Intervention Description** | **Representative Reported Outcomes and Representative Results** |
| --- | --- | --- | --- | --- |
| Boger E; 2003; USA | ED | Intervention start date: Oct 1999 | Implementation of a digital Emergency Care Tracking Board designed to improve workflow within the ED and Urgent Care, and decrease patient LOS. | INCREASE: Patient satisfaction in the ED and UC notably improved after the intervention. Post implementation, “Waiting time to treatment area” [% ranking] improved in patient satisfaction from Q1 to Q4 in the ED [75 to 85.7], and in UC [77.3 to 80]. Satisfaction of patients and families about delays improved from Q1 To Q4 in the ED [74.3 to 77.7] and in UC [70.7 to 79.5]. [Paper did not report any detailed quantitative results or statistical analyses to support this finding]. |
| Dykes PC, et al. 2017; USA | ICU (Medical) | Pre-intervention:1 Jul 2013 –  8 Jun 2014 (n=881)  Post-intervention: 1 Jul 2014 – 29 May 2015 (n=904) | Implementation of a web-based Patient Engagement Communication and Technology (PROSPECT) program with patient portal which aimed to improve patient safety and outcomes. | INCREASE: Patient satisfaction with overall hospital care improved, and care partner satisfaction with the ICU experience also improved post-intervention. Patient satisfaction [% (95% CI)] improved from [71.8 (61.1 - 82.6)] to [93.3 (88.2 - 98.4)] (**p<0.001**), and care partner satisfaction improved from [84.3 (81.3 - 87.3)] to [90.0 (88.1 - 91.9)] (**p<0.001**). |
| Hartzler, AL; et al. 2016; USA | Oncology | Total n=12 | Design and evaluation (Pilot study) of a Patient Reported Outcomes (PRO) dashboard to display trends in health-related quality of life (HRQOL) for patients treated for prostate cancer. | NONE: No clinically or practically significant change in the Patient Visit Satisfaction measured by using patient satisfaction scale (PtSS) for visits. PtSS [mean (SD)] without PRO dashboard was higher at 3-month visit [5.3 1.12)] than at 6-month visit with PRO dashboard [4.6 (1.51)].  No clinically or practically significant change of the Patient-provider Communication measured by using the Patient-doctor interaction scale (PDIS). PDIS [mean (SD)] was marginally lower at 3-month visit without PRO dashboard [86.8 (5.31)] than at 6-month visit with PRO dashboard [87.0 (4.51)]. [Paper did not report any detailed quantitative results or statistical analyses to support this finding]. |
| Nash M, et al. 2010; USA | Inpatient | Intervention start dates:  Post-discharge Calls: Sep 2008 (Q3)  Nurse Leader Rounding: Jan 2009 (Q1) | Implementation of a web-based dashboard designed to improve patient satisfaction via tracking and reporting of compliance with process measures (Post-discharge phone calls and Nurse Leader Rounding). | INCREASE: Inpatient satisfaction scores (measured on the Hospital Consumer Assessment of Healthcare Providers and Systems – HCAHPS survey instrument)  improved [F=44.8] (p=0.01) from Q3 (2006) with 56% of answers rating 9 and 10 compared to Q3 (2009) with 71% of answers rating 9 and 10. |
| Niday P, et al. 2012; USA | Hospital-wide | Pre-intervention: 2008  Post-intervention: 2009 | Implementation of a multifaceted intervention, including a financial dashboard, to improve nurse scheduling in acute care settings and reduce nurse staffing expenditure. | NONE: No improvement or deterioration in patient satisfaction during the intervention and up to the assessment of the effects of the intervention [in 2009]. [Paper did not report any detailed quantitative results or statistical analyses to support this finding]. |
| Rachmat FD, et al. 2017; Indonesia  [Conference Abstract only] | Inpatient Medicine  Data Collection Periods: | Pre-intervention: 2015  Post-intervention: 2016 | Implementation of an Advancing Bed Management Dashboard to streamline bed management and improve clarity of information between hospital units, thereby improving inpatient satisfaction. | INCREASE: Improved inpatient satisfaction from 80% to 90% (from pre- to post-intervention). [Paper did not report any detailed quantitative results or statistical analyses to support this finding]. |
| Thompson C, 2014; USA  [Conference Abstract only] | Inpatient Medicine | Data Collection Period: 10 months | Implementation of a web-based Heart-Failure dashboard as part of a quality improvement project to improve patient transitioning to home, and reduce the rate of readmission for heart failure patients. | INCREASE: Patient satisfaction related to discharge improved by 5% post-intervention compared to pre-intervention. [Paper did not report any detailed quantitative results or statistical analyses to support this finding]. |
| Wang JC, et al. 2019; Taiwan  [Conference Abstract only] | Inpatient | Intervention Period: Jan 2017 – Dec 2018  Pre-intervention: (n=10)  Post-intervention: (n=10) | Implementation of an App linked to a web-based dashboard to facilitate communication between patients and ERAS staff members and improve efficiency and efficacy of care. | INCREASE: Improved patient satisfaction post-intervention compared to pre-intervention. Patients Mean score on ‘Satisfaction Scale’: pre-intervention [6/10], post-intervention [8.5/10] (**p=0.03**). |

**eTable 7. Acronyms for Quantitative Results Tables**

| **Acronyms for Quantitative Results Tables** | |
| --- | --- |
| ADE | ADE – Adverse Drug Event |
| CA-BSI | Catheter Associated Blood Stream Infections |
| CDSS | Clinical Decision Support System |
| ED | Emergency Department |
| EHR | Electronic Health Record |
| EM | Emergency Medicine |
| EMR | Electronic Medical Record |
| EPR | Electronic Patient Record |
| ERAS | Enhanced Recovery After Surgery |
| HAUTI | Hospital-Associated Urinary Tract Infection |
| ICP | Infection Control Professionals |
| ICU | Intensive Care Unit |
| IRR | Incidence Rate Ratio |
| OR | Operating Room, or Odds Ratio (statistic) |
| OT | Operating Theatres |
| pADE | potential Adverse Drug Event |
| RRT | Rapid Response Team |
| VAP | Ventilator Associated Pneumonia |

**
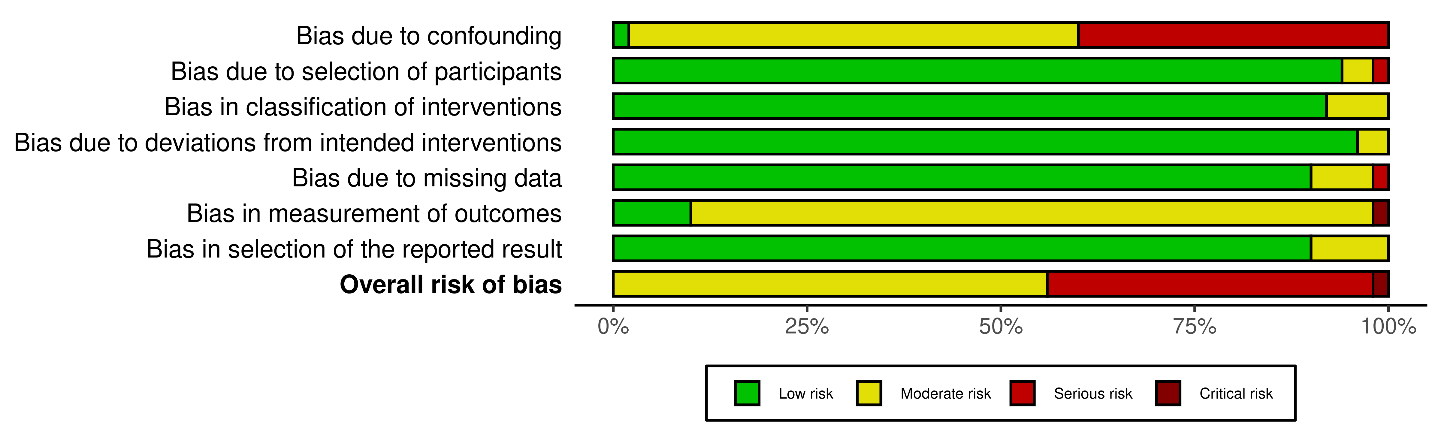
eFigure 3. Summary Plot of Risk-of-Bias Domains as per ROBINS-I Tool**

**
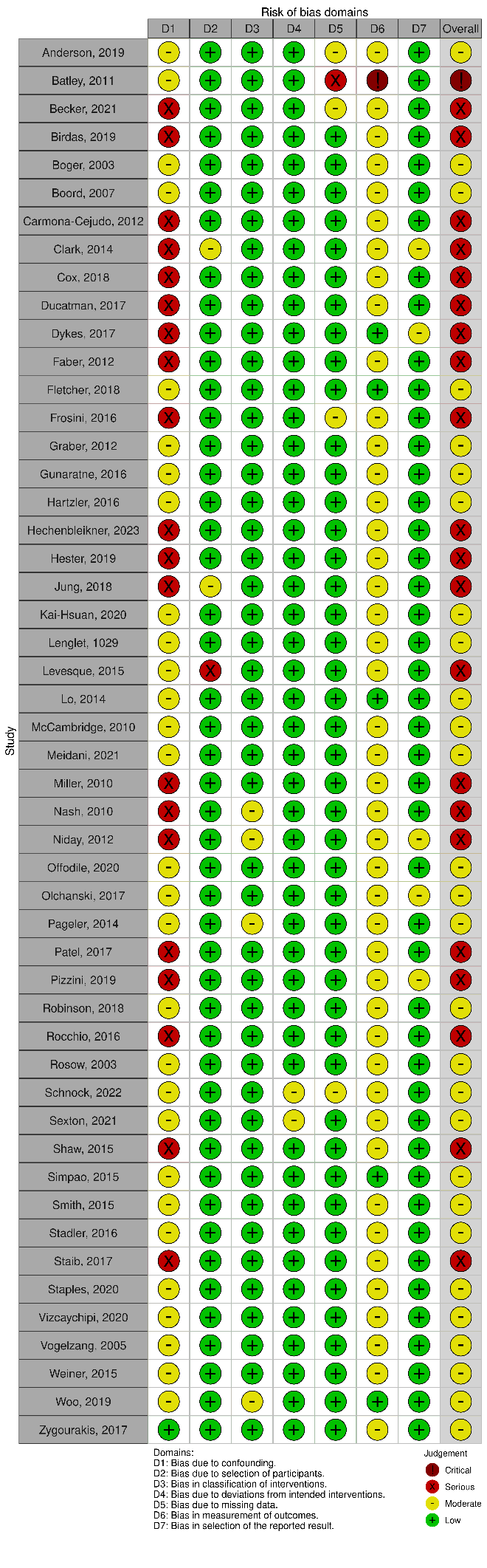
eFigure 4. Traffic Light Plot of Risk-of-Bias Domains as per ROBINS-I Tool**

**eFigure 5. Traffic Light Plot of Risk-of-Bias Domains as per Cochrane RoB 2 Tool (Individual RCT)**

**
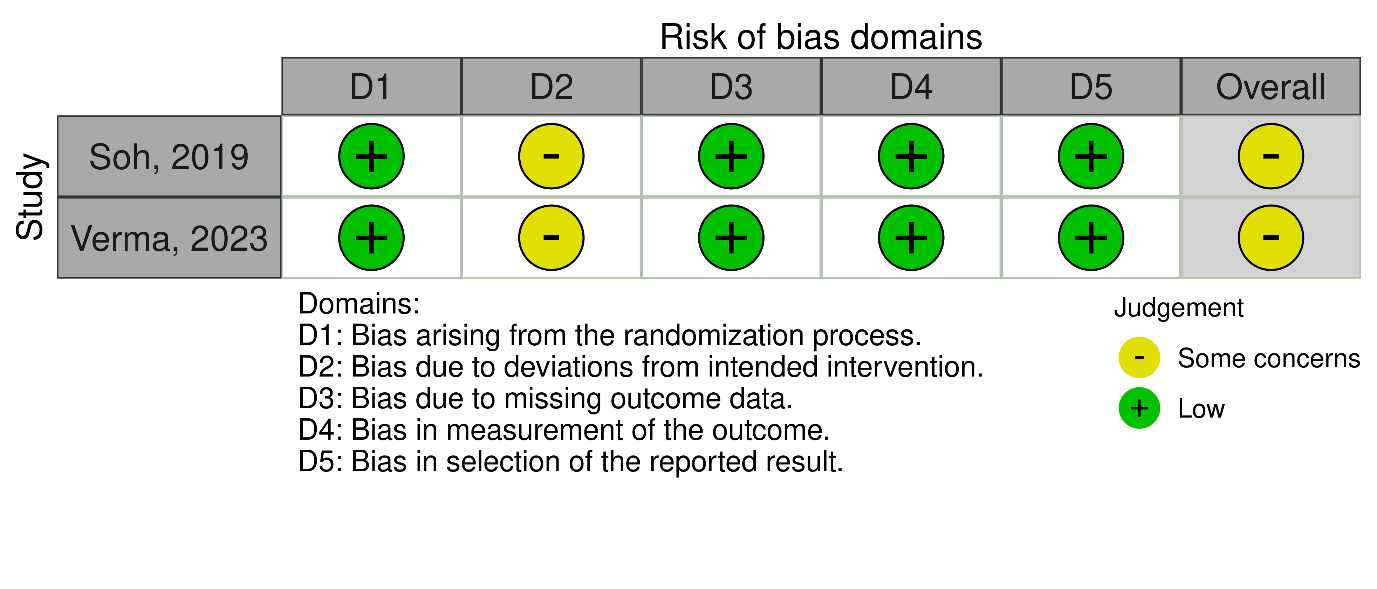
**

**eFigure 6. Traffic Light Plot of Risk-of-Bias Domains as per Cochrane RoB 2 Tool (Cluster RCT)**


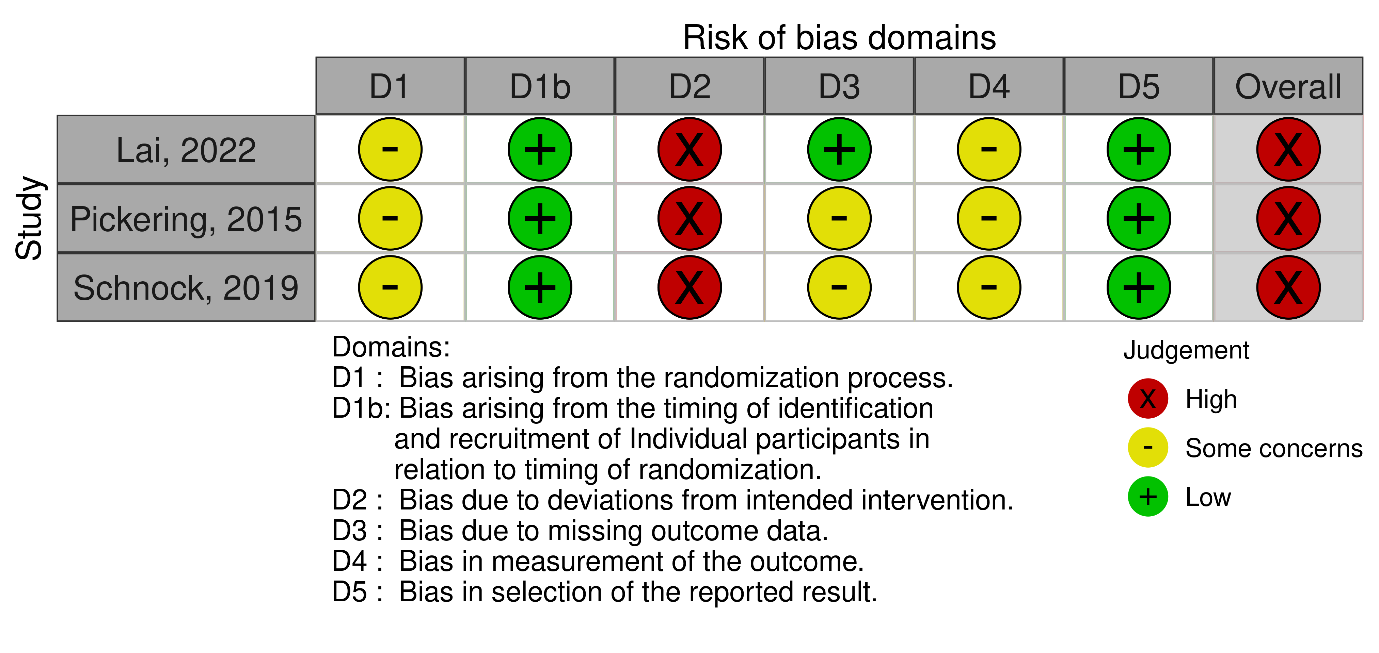


Risk-of-bias plots were generated using robvis (McGuinness et al. 2020).

McGuinness, LA, Higgins, JPT. Risk-of-bias VISualization (robvis): An R package and Shiny web app for visualizing risk-of-bias assessments. Res Syn Meth. 2020; 1- 7. <https://doi.org/10.1002/jrsm.1411>
